# Non-motor Brain Regions in Non-dominant Hemisphere Are Influential in Decoding Movement Speed

**DOI:** 10.3389/fnins.2019.00715

**Published:** 2019-07-16

**Authors:** Macauley Smith Breault, Zachary B. Fitzgerald, Pierre Sacré, John T. Gale, Sridevi V. Sarma, Jorge A. González-Martínez

**Affiliations:** ^1^Neuromedical Control Systems Laboratory, Department of Biomedical Engineering, Institute of Computational Medicine, Johns Hopkins University, Baltimore, MD, United States; ^2^Department of Neurosurgery, Cleveland Clinic, Epilepsy Center, Neurological Institute, Cleveland, OH, United States; ^3^Gale Neurotechnologies Inc., Smoke Rise, GA, United States

**Keywords:** movement speed, StereoelEctroencEphalography (SEEG), Local Field Potential (LFP), generalized linear model (GLM), regression, non-dominant hemisphere, non-motor brain regions, Least Absolute Shrinkage and Selection Operator (LASSO)

## Abstract

Sensorimotor control studies have predominantly focused on how motor regions of the brain relay basic movement-related information such as position and velocity. However, motor control is often complex, involving the integration of sensory information, planning, visuomotor tracking, spatial mapping, retrieval and storage of memories, and may even be emotionally driven. This suggests that many more regions in the brain are involved beyond premotor and motor cortices. In this study, we exploited an experimental setup wherein activity from over 87 non-motor structures of the brain were recorded in eight human subjects executing a center-out motor task. The subjects were implanted with depth electrodes for clinical purposes. Using training data, we constructed subject-specific models that related spectral power of neural activity in six different frequency bands as well as a combined model containing the aggregation of multiple frequency bands to movement speed. We then tested the models by evaluating their ability to decode movement speed from neural activity in the test data set. The best models achieved a correlation of 0.38 ± 0.03 (mean ± standard deviation). Further, the decoded speeds matched the categorical representation of the test trials as correct or incorrect with an accuracy of 70 ± 2.75% across subjects. These models included features from regions such as the right hippocampus, left and right middle temporal gyrus, intraparietal sulcus, and left fusiform gyrus across multiple frequency bands. Perhaps more interestingly, we observed that the non-dominant hemisphere (ipsilateral to dominant hand) was most influential in decoding movement speed.

## 1. Introduction

The underlying neural correlates of movement have captivated neuroscientists throughout history; however, research has typically focused on the primary, supplementary, and pre-motor cortices. Areas of the brain outside of these have been less studied or even overlooked and as a result, little is known about the extent to which these non-motor regions may play a role in motor coordination. There are at least two reasons for this gap in the literature. Movements that would largely engage non-motor regions may be more complex and thus require cumbersome experimental setups, and/or capturing activity in these regions requires a recording modality with large brain coverage and fine spatial and temporal resolution (Diedrichsen et al., 2005; Logothetis, 2008; González-Martínez et al., 2015). One setup that has been used is recording scalp EEG from humans while they execute motor tasks. For example, in Grave de Peralta et al. (2009), researchers demonstrated that they could accurately classify which hand subjects would use on a trial-by-trial basis by first estimating intracranial potentials from the scalp EEG (Grave de Peralta-Menendez and Gonzalez-Andino, 2008) and then relating high frequency spectral power of these estimated signals to hand movement intention. Scalp EEG is advantageous as it is noninvasive. However, deeper structures governing cognitive functions that must be recruited for good motor performance are inaccessible from scalp recordings. There are several approaches to estimating activity from deeper structures in the brain from scalp EEG, including source localization; but, these approaches suffer from smearing of the signals due to skull conductivity, making them less desirable than directly recording from the source.

Despite limitations in neural access, it is important to look at both cortical and subcortical areas of the brain that may be responsible for supporting motor function. In order for an action to be successfully accomplished, a goal must be established, a sequence of steps must be formulated, and a series of actions must be executed (Grafton and de C. Hamilton, 2007). Furthermore, movement must be continuously controlled throughout its performance and updated if it is perturbed. Previous research examining these components has suggested a hierarchical organization for the processes associated with movement (Grafton and de C. Hamilton, 2007). Spatial and sensory information about the external environment are integrated primarily within areas of the parietal lobe (Cole et al., 2014); specifically, the intraparietal sulcus plays a well-known role in merging perceptive and motor elements of “hand-eye” coordination (Grefkes and Fink, 2005) while the precuneus is involved in directing attention in space (Cavanna and Trimble, 2006). Fibers of these pathways are then suggested to project to motor areas of the frontal cortex (Petrides and Pandya, 1984). While regions of the temporal lobe are not typically considered to be integral modules of the motor system, several studies have implicated their role in path integration and planning, visuomotor tracking, spatial mapping, and kinematic encoding (Fyhn et al., 2004; Epstein et al., 2007; Yamamoto et al., 2014). Most notably, a study by Tankus and Fried (2012) demonstrated that medial temporal lobe structures were significantly activated during a center-out motor task, suggesting that this activity was indicative of temporal lobe involvement in the transformation of visual input to hand movement and may be connected to the dorsal pathway.

The current study seeks to explore the neurophysiological underpinnings of non-motor regions in a movement task through the process of constructing models that relate neural activity to movement speed. The models are then evaluated by assessing their ability to predict movement speed solely from neural activity in non-motor regions. We hypothesize that a simple generalized linear model structure can address whether non-motor regions can be used to accurately predict movement speed. To test our hypothesis, we exploited an experimental setup wherein eight participants implanted with intracerebral depth electrodes (i.e., StereoElectroEncephaloGraphy, SEEG), that covered several brain regions mentioned above, performed a center-out motor task that cued for various speeds. SEEG is particularly advantageous because it allows for millisecond-level recordings as well as direct access to subcortical and superficial areas of the brain (González-Martínez et al., 2015).

A generalized linear modeling framework was then used for constructing subject-specific models that predicted the movement speed on a given trial as a function of combinations of spectral features computed from SEEG recordings. Seven different models were compared that used frequency band oscillations from only (i) theta (4–8 Hz), (ii) alpha (8–15 Hz), (iii) beta (15–30 Hz), (iv) low gamma (30–60 Hz), (v) high gamma (60–100 Hz) activity, (vi) hyper gamma (100–200 Hz), and (vii) a combined model that allowed for a combination of these frequency bands (Crone et al., 1998a,b; Basar et al., 2000; Kahana et al., 2001; Gonzalez et al., 2006; Canolty and Knight, 2010). Each model was then evaluated by testing its ability to decode the movement speed from the measured neural activity.

Our preliminary results corroborate well-established phenomena and suggest other roles of non-motor regions. Specifically, we found that using a combination of frequency bands produced the most accurate estimations out of the seven possible models. Performance metrics, including correlations and errors, were computed on a test data set to evaluate the models selected from cross-validation on training data across all subjects. We also found similar regions were selected as features across subjects, including right hippocampus, middle temporal gyrus, intraparietal sulcus, and left fusiform gyrus. The functionality of these groups ranges from multisensory integration to agency. We believe we are mainly capturing regions involved with visual processing, however, interestingly our study provides preliminary electrophysiological evidence of lateralization of the non-dominant hemisphere. In particular, spectral features from the non-dominant hemisphere (ipsilateral to dominant hand) were selected more often to decode movement speed.

## 2. Materials and Methods

### 2.1. Subjects

SEEG recordings were performed in medically refractory epileptic patients for the clinical purpose of finding the Epileptogenic Zone (EZ) for possible resection (Talairach and Bancaud, 1973). This study did not alter any invasive procedure as electrode locations were made based on postoperative measurements independent of this study.

Criteria for subjects undergoing SEEG implantation were reviewed by clinicians in order to determine whether the subject would be eligible to enroll in this current study. This review process allowed for eight individuals over the age of 18 with the ability to provide informed consent and to perform the behavioral task. Subject enrollment was completely voluntary and all subjects gave informed consent. Alterations to their clinical care were not made other than the behavioral experiments. The retrospective data collection of this study was approved by the Cleveland Clinic Institutional Review Board. Table 1 contains demographic information for the subjects who participated in this study.

**Table 1:** Table of the number, demographics, handedness, and the number of trials for each subject.

**Subject**	**Sex**	**Age (years)**	**Handedness**	**Number of trials**
1	F	29	L	105
2	M	26	L	76
3	F	41	R	76
4	F	55	R	30
5	F	60	R	113
6	F	37	L	128
7	F	32	R	122
8	M	24	R	117

### 2.2. StereoElectroEncephaloGraphic (SEEG) Implantation

**Figure 1:**
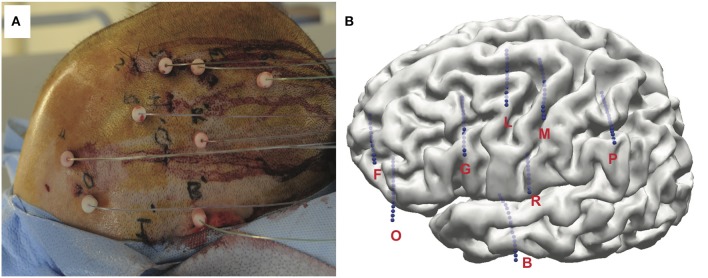
**(A)** Example of a final postoperative image of left frontal-temporal-parietal SEEG implantation. Subject shown did not perform task. **(B)** Three-dimensional MRI reconstruction showing details of subject's unique cortical anatomy and the relative position and depth of of implanted electrodes.

Implantation of the SEEG depth electrodes (PMT Corporation, MN, USA) was performed at the Cleveland Clinic using co-registered three-dimensional CT and MRI scans (González-Martínez et al., 2015). Approximately 8–13 depth electrodes were stereotactically implanted per subjects (Figures 1A,B). Along each electrode are between 10 and 16 contacts spaced 1.5 mm apart, each with a length of 2 mm and a diameter of 0.8 mm. Electrodes were inserted using a robotic surgical implantation platform (ROSA, Medtech Surgical Inc., USA) in orthogonal or oblique orientation. This allowed for intracranial recording from lateral, intermediate, and/or deep cortical and subcortical structures in a three-dimensional arrangement (González-Martínez et al., 2015). The day prior to surgery, volumetric postoperative MRIs (T1, contrasted with Multihance®, 0.1 mmol kg-1) were obtained to postoperative plan safe electrodes trajectories the day prior to the surgery. Adjustments to the insertion trajectories were made to avoid vascular structures. Each electrode contact was labeled according to the anatomical location from postoperative CT images to each subject's postoperative structural MRI scan. Following implantation surgery, a three-dimensional reconstruction of the co-registration was produced and visualized in Curry Neuroimaging Suite (Neuroscan, El Paso, USA) and the location of each contact manually determined with the agreement of at least two clinical experts (Figures 1C,D). Electrode placement, EZ, and electrode coverage for each subject can be found in Table S1 and Figure S1.

### 2.3. Electrophysiological Recordings

Neural recordings of Local Field Potential (LFP) activity–from superficial to deep non-motor brain structures–were collected onsite at a sampling rate of 2 kHz using clinical electrophysiology acquiring system activity (Nihon Kohden 1200, Nihon Kohden America, USA) in the Epilepsy Monitoring Unit. The recording sessions used for this study were free of epileptic seizures.

### 2.4. Motor Task

**Figure 2:**
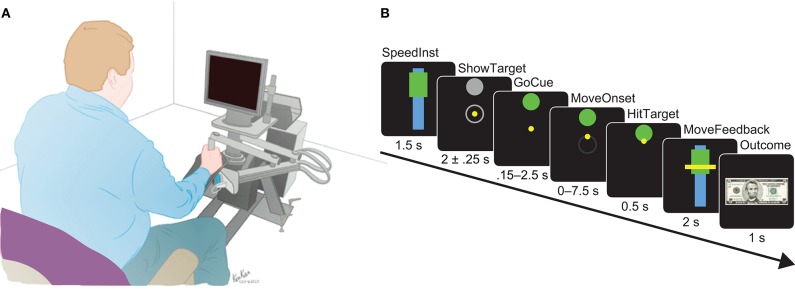
**(A)** Apparatus set-up of the robotic manipulandum and attached screen, where the visual stimuli for the motor task were displayed. This system allowed for precise tracking of arm movements over a horizontal plane as the subjects controlled a cursor shown on the screen with the task stimuli. **(B)** Detailed timeline of epochs during a single trial shown using simulated screens. The interval of time in which the stimuli were presented are shown below each simulated epoch. Models were built using the neural activity recorded between MoveOnset and HitTarget.

Subjects performed goal-directed reaching movements with speed instructions that have been previously described (Johnson et al., 2014; Breault et al., 2017, 2018, 2019a,b; Kerr et al., 2017). Movements were made using a robotic manipulandum from the InMotion ARM Interactive Therapy System (Interactive Motion Technologies, Watertown, MA, USA) and were displayed as a cursor on an attached computer screen (Figure 2A). Subjects used their dominant hand to control the robotic arm, with handedness listed in Table 1. The interface was prepared in MATLAB®(Mathworks, Natick, MA) using MonkeyLogic (Asaad and Eskandar, 2008).

The goal of the task was to move a cursor to the designated target at an instructed speed. Speeds were relative to each subject based on calibration trials of their fastest movements prior to testing. The task was broken down into several epochs, as shown as simulated screens in Figure 2B. One session consisted of 120 trials. Each trial began with a speed instruction designated by the placement of the rectangle relative to the bar; high for fast and low for slow. The instructed speed is displayed for 1.5 s (SpeedInst). The fixation epoch was presented in which the subject was expected to move the yellow cursor to the center within 7.5 s. After the cursor was in the center, the target was revealed in one of four locations (ShowTarget): right, left, up, or down. After a delay period of 2.00 ± 0.25 s, GoCue began when the target changed color from gray to green. The subject was expected to initiate their movement by moving the cursor within 0.15–2.5 s (MoveOnset) of GoCue or else the trial would forfeit. After movement onset, the subject had a maximum of 7.5 s to move the cursor to the appointed target. The movement was considered complete once the subject held the cursor in the target for 0.50 s (HitTarget). After movement completion, their speed was calculated and the ratio relative to their calibration speed as found. This was then compared to the instructed speed, where their speed must fall between 66.7 ± 13.3% of their calibrated speed for fast trials or between 33.3 ± 13.3% of their calibrated speed for slow trials. Their actual speed was shown as visual feedback relative to the instructed speed by the placement of a horizontal line across the speed bar and instructed speed rectangle for 2 s (MoveFeedback). Finally, an outcome was displayed for 1 s indicative of whether they correctly met the instructed speed or not: either a $5 bill (Reward) or a red “X” over the $5 bill (SpeedFail). Perturbations with random magnitudes and directions were applied via the robotic arm immediately entering movement onset to 20% of the trials. Only non-perturbed trials were considered for the purposes of this study.

### 2.5. Data Preparation

The raw electrophysiological and behavioral data were preprocessed in preparation for model fitting as described below. Some subjects performed the motor task over multiple sessions. In those cases, we chose to only model the first session as a representative for those subjects.

#### 2.5.1. Neural Data

The neuronal activity from the SEEG electrodes was preprocessed using spectral analysis. First, the data were filtered using a Notch filter with a notch located at the fundamental frequency of 60 Hz and the bandwidth at the -1 dB point set to 3 Hz. Next, oscillatory power was calculated using a continuous wavelet transform with a logarithmic scale vector ranging 1–200 Hz and complex Morlet wavelet with a default radian frequency of ω_0_ = 6. Using a time window of 100 ms every 50 ms, the instantaneous power spectral density was divided and averaged over each overlapping time bins (50%) where each 100 ms time bin was labeled using the last temporal index corresponding to that window. Finally, the averaged power spectral density was normalized in order to equally weigh all frequencies. This was done by taking the standard score (*z*-score) of the natural logarithm of the power in each frequency bin over the entire recording session time. All contact recordings were visually inspected for artifacts. Channels with imperfections such as broadband effects or abnormal bursts of power were disregarded for all trials.

The result of preprocessing was spectral data for each electrode contact over a range of frequencies for the entire session. These data were used to calculate features that represented neural activity in the decoding model. Features were calculated by averaging the neural activity of each electrode contact in the spectrogram over a range of frequency bins around a window of time-related to an epoch for each trial. Each feature is associated with an electrode contact, label, frequency band, and time window.

A time window between MoveOnset and HitTarget was chosen to capture the neural activity modulating during movement. We used the following ranges of frequencies commonly referenced in literature to divide the data into bands: theta (4–8 Hz), alpha (8–15 Hz), beta (15–30 Hz), low gamma (30–60 Hz), high gamma (60–100 Hz), and hyper gamma (100–200 Hz).

#### 2.5.2. Behavioral Data

Prior to the task, subjects underwent a calibration epoch where they were instructed to make 20 fast movements to the right. Their fastest movement speed was recorded and used to calibrate trial speeds for the remainder of the session. Trial speed was computed by dividing the constant length of the straight line between the center of the center and the center of the target by the time between MoveOnset and the final HitTarget for each completed trial. The acquisition system then calculated the ratio of this trial speed to their calibration speed. This meant that the speed of each trial was saved as a value between [0, 1], where 0 represents a trial where the subject did not move and 1 represented a trial in which the subject reached a trial speed greater than or equal to their calibration speed.

**Figure 3:**
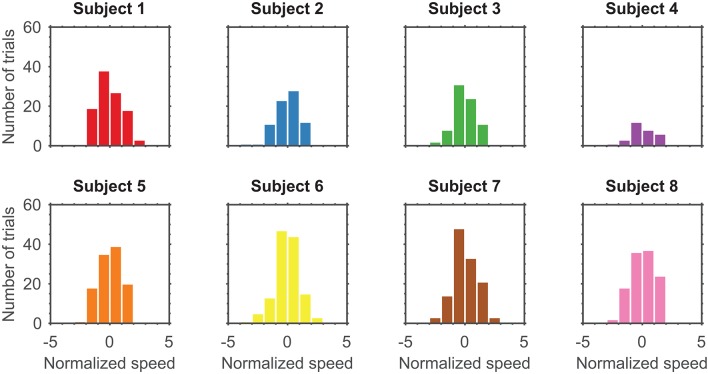
Distribution of normalized speeds across all trials for eight subjects with a bin width of one.

Prior to modeling, speeds were normalized to fit a normal distribution by taking the standard score of the natural logarithm of speeds from all completed, unperturbed trials over the entire session (Figure 3). This left an average of 95.88±33.09 (mean ± standard deviation) trials for modeling. Let *T*_*n*_ denotes the total number of trials available to build a model for subject *n*. Refer to Table 1 for the exact number of trials used for modeling each subject.

### 2.6. Decoding Model and Procedure

All electrophysiological modeling and decoding analyses were conducted offline using custom MATLAB®scripts. Decoding is formulated as a linear regression model, in which there exists a linear relationship between the dependent variable, *speed*, and the independent features consisting of the *neural activity* from the electrode contacts over various frequency bands (Holdgraf et al., 2017).

We set out to answer whether neural activity could predict the speed of each movement. We hypothesized that there exists a relationship between the neural activity in non-motor brain regions during movement and the speed of the trial. We tested this hypothesis by first constructing decoding models developed purely on a training set and then applying these models to decode speed on a trial-by-trial basis on a test set. Specifically, we split the data using 80% of the trials for the training set and the remaining 20% of the trials for the test set. The training set was used to extract features and train the linear regression model. We then applied this model to the test set to decode speed trial-by-trial. Features were extracted using a data-driven approach that optimized the hyper-parameter over cross-validation on the training set. The modeling framework is described below.

#### 2.6.1. Modeling Speed as a Function of Neural Activity

We constructed subject-specific models to eventually be used to predict speed from the neural activity across multiple electrode contacts and frequency bands. In particular, we assumed that the normalized speed of subject *n* on trial *t*, denoted *y*_*n*_(*t*), is a random variable with a Gaussian distribution whose mean depends on a feature vector ${\text{x}}_{n}\left(t\right)\in {\mathbb{R}}^{J_{n}}$ containing *J*_*n*_ features of neural data that from regions that encode speed. The mean of the distribution is modeled as follows:

(1) $$\begin{array}{r} E[y_{n}(t)\;|\;x_{n}(t)]=\beta_{n0}+\sum_{j=1}^{J_{n}}\beta_{nj}x_{nj}(t), \end{array}$$

where β_*n*0_ is a constant representing baseline speed and β_*nj*_ is a coefficient that weighs the influence that feature *j* of subject *n*, denoted *x*_*nj*_, has on the movement speed.

#### 2.6.2. Feature Selection and Model Fitting

Spectral power of neural activity in certain frequency bands has been shown to encode and communicate information at a population-level in the brain (Ward, 2003). Therefore, we constructed seven models differentiated by features capturing the spectral content of neural activity in different frequency bands. Specifically, the models that were compared used either (i) theta band features (4–8 Hz), (ii) alpha band features (8–15 Hz), (iii) beta band features (15–30 Hz), (iv) low gamma band features (30–60 Hz), (v) high gamma band features (60–100 Hz) activity, (vi) hyper gamma band features (100–200 Hz) activity, or (vii) a combination of all frequency bands (Crone et al., 1998a,b; Basar et al., 2000; Kahana et al., 2001; Gonzalez et al., 2006; Canolty and Knight, 2010). To compute features from different frequency bands, we computed a spectrogram for each electrode contact and each trial. The average neural activity within each frequency band was treated as an independent feature that could be incorporated into a model.

In our data set, there were often more possible features than trials to train on. Therefore, it was essential to limit the number of features for each model. Picking the number of features to use is a balancing act. On one hand, having too many features is not only computationally expensive but also produces models that are overfitted and poorly generalizable (Guyon and Elisseeff, 2003; Pereira et al., 2009; Holdgraf et al., 2017). On the other hand, models built with too few features could lead to poor performance. The goal of feature selection for regression models is to then pick as few as possible informative features without being influenced by *a priori* knowledge.

For these reasons, we utilized a data-driven approach to select a subset of features for each model. Features were selected using the Least Absolute Shrinkage and Selection Operator (LASSO) method. This method attempts to minimize the errors using regularization, which penalizes the number features with non-zero coefficients in the model. Therefore, it drives the coefficients of the uninformative features in the regression model. It is formally defined as the solution to the *l*_1_ optimization problem (Tibshirani, 1996):

(2) $$\begin{array}{r} \underset{\beta }{\min}∥y-X\beta {∥}^{2}\text{ subject to }∥\beta {∥}_{1}=\sum_{j=1}^{J_{n}}|\beta_{j}|⩽\lambda , \end{array}$$

where 0 ⩽ λ is the hyper-parameter that controls the penalty factor, where large values of λ drive more coefficients to zero.

Specifically, a 10-fold cross-validation procedure was performed on the training set to select features by grid searching across all values of λ. The goal was to find the optimal hyper-parameter $\left(\hat{\lambda }\right)$ that minimizes the Mean Squared Error (*MSE*). The procedure began by dividing the training set into 10 subsets of approximately equal size. In each fold, 9 out of the 10 subsets were used to train models over gridded values of λ using the lasso function in MATLAB®. Essentially, the output of LASSO is then a vector of coefficients on the feature matrix for every value of lambda. These fitted models were then used to estimate the speed on the single subset of trials that were not used for training. This subset is known as the validation set. The *MSE* between the estimated speeds the actual speeds of the validation set was calculated for every model associated with a λ. This process was repeated such that every subset was used for validation. At the end of cross-validation, the average error *MSE* across all folds was calculated for each hyper-parameter value and $\hat{\lambda }$ was chosen to minimize this error.

Finally, the final subset of features was obtained by running the entire training set through LASSO and selecting the features with non-zero coefficients at $\hat{\lambda }$. To assess the stability of these features, the entire cross-validation procedure was repeated for 100 different splits of the training set, keeping the test set the same. Once the model building procedure was validated and the features were selected, a final model was constructed using glmfit function in MATLAB®on the training set.

#### 2.6.3. Evaluating Model Performance

Once a model was constructed via the aforementioned procedure on training data we evaluated the performance of the said model by assessing its ability to decode movement speed using neural activity on a trial-by-trial basis in the test set. Keep *t* with trial, the model was used to predict the trial speed for subject *n*, denoted as ŷ_*n*_(*t*), using Equation 1. To evaluate the performance of a fitted model on the test set, we used the Pearson correlation coefficient (*R*) and *MSE* to measure goodness-of-fit.

#### 2.6.4. Visualizing Feature Maps

To intuitively understand the range of the significance of the features found using our analysis, we chose to highlight them using a human MRI atlas (Mori et al., 2005). This was done by mapping features from the model to atlas labels. Each feature represents the neural activity of a physical electrode contact with an anatomical label prepared by clinicians (section 2.2). These feature labels were then matched to labels from the Mori atlas and template MRI. The matched labels were subsequently highlighted on the template MRI in the representative subject color. Despite the fact that each electrode contact may have only recorded from a small portion of a brain region, it is impossible to verify the consistency of mapping the electrode coordinates to the atlas coordinates across subjects. Therefore, we chose to highlight the entire brain region on the template MRI to visualize coverage across subjects, as opposed to highlighting smaller sections of a region. Approximately 16.36% of the labels could not be matched to the atlas, which namely consisted of sulci and opercula. These labels were disregarded for the purposes of mapping.

To demonstrate the stability of the features for each subject, we used a tinting system in which each highlighted region was tinted based on the number of times that region was selected as a final feature across all 100 iterations of the cross-validation procedure discussed in section 2.6.2. Brain regions that were selected as final features more often were tinted a darker color and regions that were selected less often were tinted whiter. Sagittal, axial, and coronal slices were chosen to maximize the number of highlighted regions shown. Contacts not chosen as a feature were hatched.

## 3. Results

Our proposed model building procedure was evaluated for generalizability and producing accurate predictions on eight independent subjects (*N* = 8) consisting of behavioral and neural data. After training a final model using the features selected at the optimal hyper-parameter found from cross-validation on the training set, we evaluated the predictive power of the final model on the unseen test set. Here, we report the results of this final model for feature selection on the combined neural activity from all available brain regions across all frequency bands. The results for the other models (theta, alpha, beta, low gamma, high gamma, and hyper gamma) are available in the Supplementary Materials.

### 3.1. Model Performance

**Figure 4:**
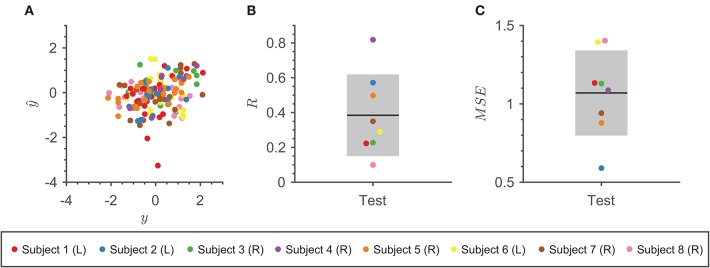
Model Performance. **(A)** Scatter plot of actual speed vs. predicted speed using the final combined model with the highest *R* for each subject over 100 iterations on the test trials. Each subject is denoted as a different color point. **(B)** The average *R* between the actual speed and predicted speed using the final combined models across 100 iterations on the test set for eight subjects. Each subject is denoted as a different color point. The mean of the metric is represented by the black line and one standard deviation is represented by the gray rectangle. **(C)** The average *MSE* between the actual speed and predicted speed using the final combined models across 100 iterations on the test set for eight subjects. Each subject is denoted as a different color point. The mean of the metric is represented by the black line and one standard deviation is represented by the gray rectangle.

Figure 4 summarizes performance metrics from cross-validation for the combined models. Despite the limited number of trials for each subject in our data set, all subject-specific models performed well. Figure 4A shows a positive linear relationship between the actual and predicted speeds over the trials in the test set. The performance of the final models found across all 100 iterations had an average $\bar{R}=0.38\pm 0.03$ and average $\bar{MSE}=1.07\pm 0.09$ across all subjects (Figures 4B,C). The best model achieved the highest *R* = 0.82 and lowest *MSE* = 0.36 over the test set.

To get an idea of how well our decoder performed under the task condition of reaching a speed within the instructed range predicted speeds were transformed back into the original [0, 1] range using the mean and standard deviation from the distribution of the trial speeds. Then, the transform speeds were converted into categorical representations of correct or incorrect by applying the speed instruction per trial and compared to the original outcomes. Our decoding model achieved an average accuracy of 70.00 ± 2.75% across iterations and subjects, which is above chance (50%). Our findings suggest that these models capture a relationship between the neural activity in non-motor brain regions and speed.

The performance of the remaining six models is demonstrated in Figure S2. By comparison, all models had roughly the same amount of error but the combined model had the highest average correlation, meaning the combined model better predicted speed over models based on a single frequency band. This was expected as we believe that the combined model would pick the most influential features from each frequency band (Figure S3).

### 3.2. Stability Analysis of Model Features

To reinforce the results of our final model, a stability analysis was performed to show that the features found during cross-validation largely overlap with the final features. Features that were frequently selected during cross-validation and also appeared in the final model play a consistent role in the model for decoding speed. Since electrode locations are not equivalent, we refer to the consistency of subjects sharing a feature with the same label as the feature being stable.

**Figure 5:**
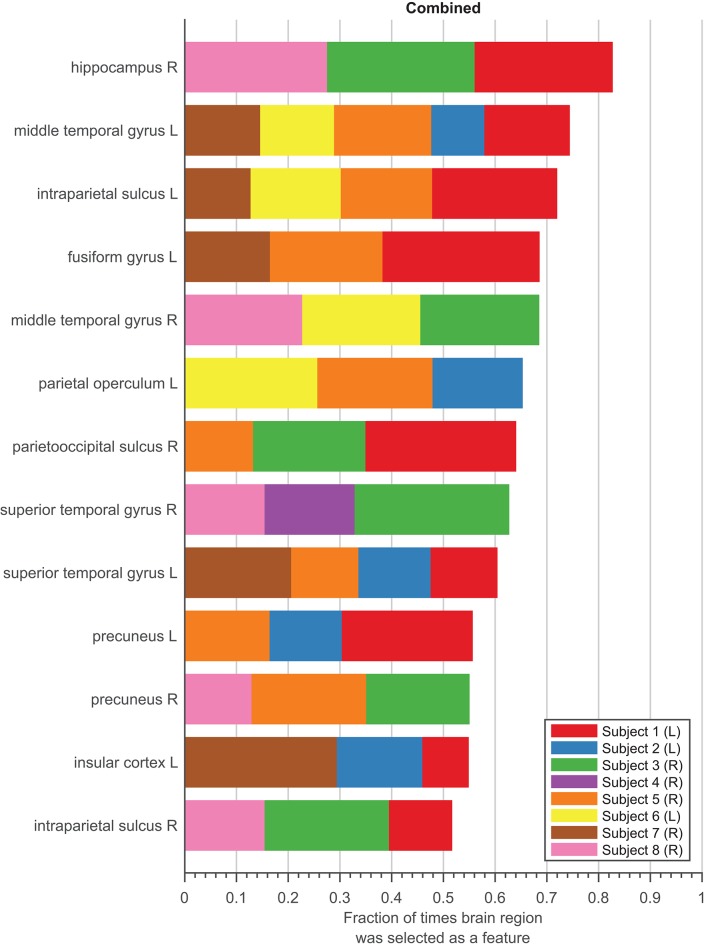
Stability Analysis. Stability of features in the combined models for eight subjects, sorted by the fraction of times feature was picked during feature selection relative to the 100 iterations during 10-fold cross-validation. Each subject is denoted as a different color bar. Length of each colored bar represents the fraction of times feature was selected relative to the total number of times feature was sampled within each subject. Only features that were selected as a final feature are shown.

We represent stability in Figure 5 as the union of features from the combined models selected during cross-validation and the final subset of features. Only features that were represented in *at least three* subjects were considered. The length of each colored section of the bar was calculated as the relative number of times the brain region was selected as a feature across all folds and iterations for each subject. The color of each section of the bar distinguishes each subject. The total length of the bar represents the relative number of times a brain region was selected, where a fraction of one would indicate that the brain region was selected in every fold for every subject that had recordings from that region across all iterations. Therefore, a feature with a longer bar can be interpreted as being selected more consistently during cross-validation. In other words, this feature is considered more stable since it is more consistently selected to decode speed no matter how the data is spliced.

Ideally, stable features should appear with a proportion closer to one and be selected by all subjects who have electrodes in the same locations. The results of the stability of the combined model are encouraging. Some variability in features was expected, as electrode contact locations were not globally consistent across subjects (Table S1). The top two features were selected for at least four subjects, meaning that these features are able to decode speed even in completely different data sets. For example, one of the top feature, middle temporal gyrus L (L = Left hemisphere), was identified as a feature for five subjects. Considering there are eight subject, this region may not appear to be stable across a population. However, only five of the eight subjects had an electrode in this region (see Figure S1). Therefore, our model selected this as a feature for every subject had a contact in this region. The middle temporal gyrus L is also selected consistent, appearing in 74.32% of the times it was sampled over 100 iterations.

Since the combined model contains features from all frequency bands, we were also interested in whether any one particular frequency band was selected as a feature more often to decode movement speed. Figure S3 shows the breakdown of the features split into the frequency bands each feature represents. Out of all the features selected, features from the beta band consisted of 22.29% of the total features, followed by theta band at 21.08%, alpha band at 19.88%, and low gamma at 17.16%. The least prevalent bands were high and hyper gamma, consisting of only 10.46 and 9.13%, respectively. These results are consistent with the frequency band models, in which fewer features were needed for the models with frequencies found more often in the combined model.

The conclusion we can draw from Figure 5 is that the features in the final combined model are relatively stable, meaning these regions consistently decode speed across a population, represented by our subjects. For the stability results of the remaining six models, refer to Figure S4.

### 3.3. Feature Analysis of Final Model

Since the electrodes were exclusively implanted in brain areas outside the primary sensorimotor system for all subjects, this data set provided the unique opportunity to study whether non-motor brain regions can decode movement characteristics such as speed. After confirming the stability of model features during cross-validation, we analyzed the features selected for the final combined model fitted to the training set used for the stability analysis. For this, we visualized the selected features on an MRI and varied the shading based on the number of times the feature was selected.

**Figure 6:**
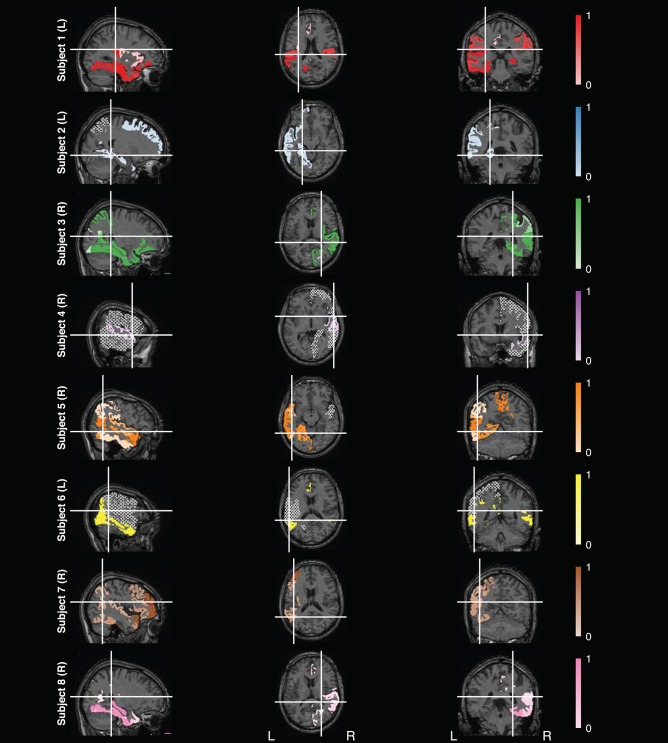
Feature Analysis. Map of features selected for the final combined model found using the test data for eight subjects, with subject numbers labeled vertically and handedness in parentheses. The white lines in each slice represents at which slice in the other viewpoints were taken. Electrode contacts selected as features were matched to labels from the Mori atlas (Mori et al., 2005). Brain regions that matched the feature labels were then highlighted on the corresponding MRI template used by the atlas. Regions were tinted based on how frequently it was selected as a final feature over 100 iterations, where regions close to zero (i.e., rarely selected as a final feature) are tinted whiter and regions close to one (i.e., often selected as a final feature) retain the original color. Not all features could be matched to an atlas label. Regions not selected as a final feature are filled in using a hatched gray area. These maps do not represent fMRI signals nor do they represent the exact location of the electrodes.

Figure 6 summarizes the non-motor brain regions that were selected as features in the final combined model. These regions were found by matching the labels of the electrode contacts for each feature with labels for an MRI atlas labels (Mori et al., 2005). However, some electrode contact could not be matched to the atlas. Note that these plots do not represent fMRI signals nor do they represent the precise location of the electrode. Regions on the MRI were tinted based on a mapping of the fraction of times feature was selected in the final combined model over 100 iterations, where lighter regions represent less informative features. Refer to Figures S5–S10 for the feature maps of the other six models.

Overall, there was variability in the features selected across subjects (Figure 5). A majority of the variability is due to the non-uniform placement of the electrodes across subjects. The discrepancy could also be caused by the subject-specific model fitting process. Subjects with fewer trials are more prone to overfitting and will not have as many features as other subjects.

## 4. Discussion

In this study, we sought to explore whether there exists a relationship between non-motor regions (outside the primary sensorimotor pathways) and movement in our SEEG data set. Linear decoding models were constructed from the data to find the relationship between the movement speed as a function of spectral content in measured neural activity during movement. We chose seven types of models based on neural activity power in specific frequency bands (theta, alpha, beta, low gamma, high gamma, hyper gamma, and combined) to study using recordings from eight independent subjects. The final model using a combination of features from different frequency bands had the highest correlation and one of the lowest errors of all seven models. We found speed encoding brain regions consistent with current knowledge as well as evidence that regions in the non-dominant hemisphere are significantly involved.

Our results suggest that brain areas encoding movement speed represent the neural correlates of cognitive components throughout the sensorimotor pathway. While the primary areas selected by the model–right hippocampus, left and right Middle Temporal Gyrus (MTG), left and right IntraParietal Sulcus (IPS), and left fusiform gyrus have been implicated in a wide variety of tasks, we believe that their appearance in the current study may further add evidence to their role in motor control.

The role of the parietal lobe structures in the sensorimotor pathway has been extensively documented in both human and non-human primates through lesion and imaging studies (Freund, 2001). The IPS, in particular, has demonstrated its involvement in spatial cognition and the integration of multisensory stimuli (Grefkes and Fink, 2005; Gottlieb, 2007; Sack, 2009). In the motor task at hand, the subject sees the target at a random location on the screen and must navigate their cursor to that location while regulating their speed in accordance with their initial instructions. By this process, the subject must direct and keep their attention on the location of the goal in order to create an internal mental representation of a path while transferring that representation to external action and object (i.e., the cursor) and maintaining their intended course.

The suggested involvement of parietal structures by our model is consistent with previous visual search studies in non-human primates. These have shown how neurons of the right inferior parietal lobe and the surrounding area are activated in the process of bringing and holding task-related information to attention (Gottlieb, 2007). While the establishment of a path toward a goal (i.e., motor planning) occurs primarily before the onset of the movement and is outside the scope of this current study, adherence to this path is monitored throughout the course of movement by continuously updating eye movement and the spatial location of the object under manipulation.

This continual update of visuospatial information and integration into external action is mirrored in a preliminary study by Papadopoulos et al. (2018) that suggests evidence of the IPS as a “general purpose,” non-context dependent mediator in object-based visuospatial transformation. The “transformation” in our current center-out task is represented by the interpretation of momentary visual information (i.e., object/cursor location) with relation to the final goal and transference of this information into regulated external action (i.e., movement that is either fast or slow). It is also indicated that the connectivity of substructures in the IPS to the occipital cortex, adjacent parietal structures, and frontal networks associated with action afford the IPS a multicomponent-integration role in the early stages of the overall sensorimotor-pathway as it relates to hand-eye coordination (Culham and Valyear, 2006). In this manner, the IPS is then responsible for directing neural information from multiple integrative interfaces to other areas of the brain for further processing and interpretation.

While the exact function of the MTG is not entirely agreed upon, it has been undoubtedly shown in the processing of semantic cognition and other comprehensive functions related to language (Binder et al., 2009). Hoffman et al. (2012) expanded upon this notion, demonstrating a functional role of the MTG in non-verbal semantic processing as well. A separate study has also suggested an ancillary role in regard to discerning discrepancies in movement tasks and intersensory conflict (van Kemenade et al., 2018). This study also proposed that the MTG (along with the angular gyrus) might be important in the process of action-feedback monitoring by establishing a sense of agency over a given action. The significant selection of the MTG by the models may reflect internal interpretation, discrimination, or maintenance of the “fast” or “slow” instruction given. Providing some overlay with theories of semantic cognition, it is possible that the activity change in MTG in our movement task may be responsible for transferring the position of the bar (top or bottom) into its meaning within the context of the task (fast or slow, respectively). It is also possible that this MTG activity relates to the monitoring of the speed of the cursor and ensuring that adherence to the correct speed instruction is preserved. Although extrapolative, considering the role of the MTG in detecting agency toward actions, we suggest that the measured activity might the active attribution of one's agency onto the cursor itself. Because the movement of the cursor is not directly a part of the participant but is nonetheless under their control, an implicit association must be made in order to establish that it is an extension of one's own movement. However, given that the MTG is more heavily activated in the detection of agency-violations and that a mismatch manipulation was not included in the study, this claim necessitates significant follow-up.

The selection of the fusiform gyrus in this context seems initially surprising, as the area is typically associated with functions of visual processing (Weiner and Zilles, 2016). Because of this, we believe this significant involvement in encoding speed is likely related primarily to the visual component of the task. Although, it is interesting to consider that the fusiform is located in close proximity to the parahippocampal gyrus, a structure whose function in spatial navigation tasks is well-known (Epstein et al., 2007). Though, a similar study on this data set has also implicated the involvement of the fusiform gyrus to speed (Breault et al., 2017).

Another important observation worth mentioning is that the model primarily selected structures of the non-dominant hemisphere in all but two of the subjects, implicating a lateralized encoding of movement speed. A thorough review by Mutha et al. (2012), notes lateralization of movement-related mechanisms into the right and left hemispheres and behavioral differences between the dominant and non-dominant arm but does not explicitly mention how these differences may carry over to hemisphere dominance, when this may be variant across individuals. Nonetheless, the authors suggest that the left hemisphere is primarily utilized in the learning of new sequences subserved by the ability to plan actions and that the right hemisphere are important for updating actions and stopping at a goal position. If the mechanisms observed in this article follow lateralization effects the same way that other highly lateralized cognitive functions (like language) do, then the preponderance of non-dominant structures in our study may be representative of the action-feedback monitoring activity component mentioned above and the target/goal-oriented nature of the motor task. One exception to lateralization is the finding of the right hippocampus as the top stable feature in both left- and right-handed subjects. The right hippocampus is thought to be more heavily involved the binding of visuospatial features and active maintenance of spatial information than the left (Piekema et al., 2006). It is important to note that 6 out of 8 of our subjects were implanted in their non-dominant hemisphere. Our results suggest that non-dominant regions are encoding speed. However, we cannot make any statements about the role of dominant hemisphere play in speed encoding due to our sampling bias.

The suggestions relating to the functional mechanisms of these brain regions cannot be taken as much more than tentative speculation based on the current literature. Further research to validate the usefulness of the subject-specific models and to elucidate the particular roles of these brain regions during movement would require individual manipulation of the cognitive variables to examine if the models generated similar results. Furthermore, the models can only select the structures that are directly sampled and cannot extrapolate beyond them, bringing into question whether or not the same areas would be selected to equal degrees across all subjects. Finally, we believe that the recorded activity originates from a localized area surrounding each contact, and is not a byproduct of volume conduction from low frequency activity generated by neighboring motor regions due to the high resistance by the extracellular medium (Grave de Peralta-Menendez and Gonzalez-Andino, 2008).

To conclude, we successfully built a model that decodes speed from non-motor regions. The results of this preliminary exploration are in line with current literature and propose a re-examination of non-motor brain regions and their role in motor control. In the future, we would like to elaborate on the model proposed here. One variation could be on either the feature vector, such as using the neural activity of other epochs such as planning period or adding complexities to features such as information from previous trials or trying other feature selection methods such as wrappers (Guyon and Elisseeff, 2003; Pereira et al., 2009).

## Ethics Statement

This study was carried out in accordance with the recommendations of Cleveland Clinic Institutional Review Board with written informed consent from all subjects. All subjects gave written informed consent in accordance with the Declaration of Helsinki. The protocol was approved by the Cleveland Clinic Institutional Review Board.

## Author Contributions

JG was involved in project development and experimental setup. JG and JG-M were involved with data collection. MB and PS were involved with data preprocessing. MB, ZF, PS, SS, and JG-M were involved with data analysis and interpretation. MB, ZF, PS, and SS were involved in manuscript preparation.

### Conflict of Interest Statement

The authors declare that the research was conducted in the absence of any commercial or financial relationships that could be construed as a potential conflict of interest. The handling editor declared a shared affiliation, though no other collaboration, with several of the authors ZF and JG-M at time of review.
