# Supplementary material for: Non-motor Brain Regions in Non-dominant Hemisphere Are Influential in Decoding Movement Speed
Source: Front Neurosci. 2019 Jul 16;13:715. doi: 10.3389/fnins.2019.00715 (PMC6660252; doi:10.3389/fnins.2019.00715)
Supplement: Supplementary file 1 [file Data_Sheet_1.pdf]

Supplementary Material: Non-motor brain regions in non-dominant hemisphere are influential in decoding movement speed

1 SUPPLEMENTARY TABLES AND FIGURES

1.1 Tables

Subject	*Handedness*	*Duration*	*EZ*	*Electrode coverage*
1	L	23	Left temporal	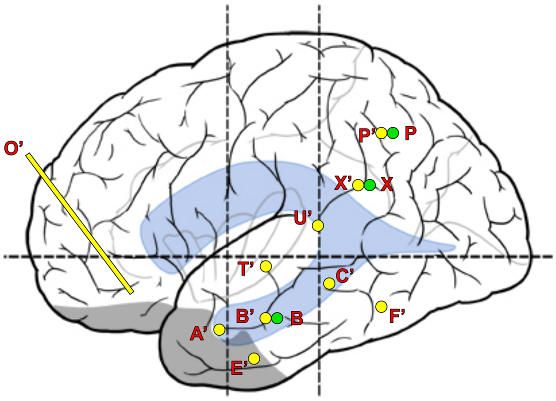
2	L	3	Left hippocampus	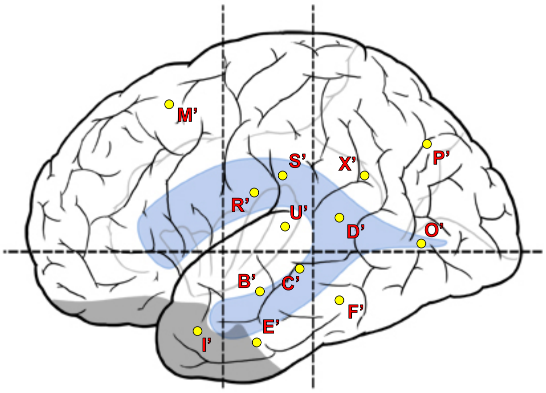

<table>
<tr><th>Subject</th><th>Handedness</th><th>Duration</th><th>EZ</th><th>Electrode coverage</th></tr>
<tr><td>3</td><td>R</td><td>38</td><td>Right hippocampus</td><td>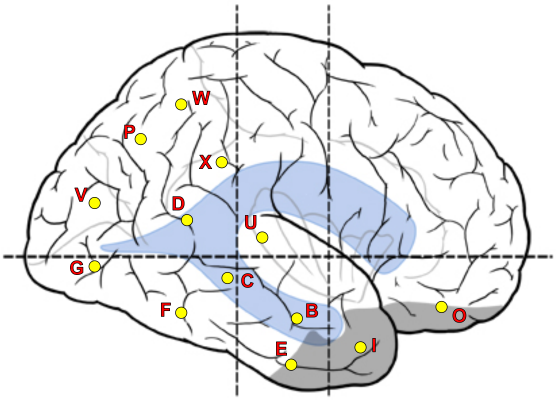
</td></tr>
<tr><td>4</td><td>R</td><td>52</td><td>Right central sulcus</td><td>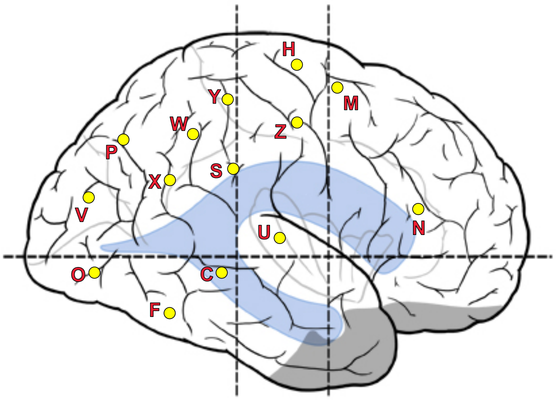
</td></tr>
<tr><td>5</td><td>R</td><td>9</td><td>Left temporal</td><td>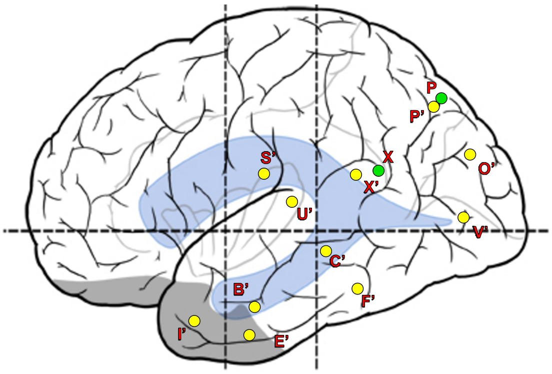
</td></tr>
</table>

Subject	*Handedness*	*Duration*	*EZ*	*Electrode coverage*
6	L	12	Left temporal	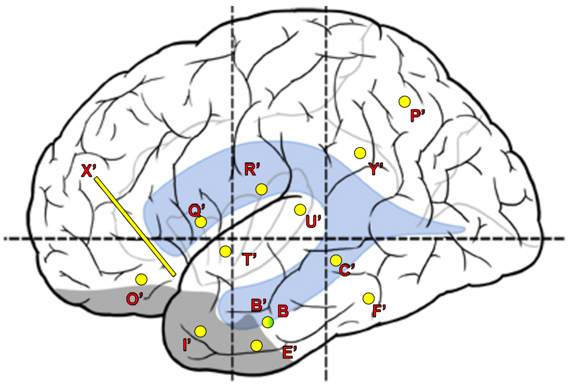
7	R	13	Left parietal	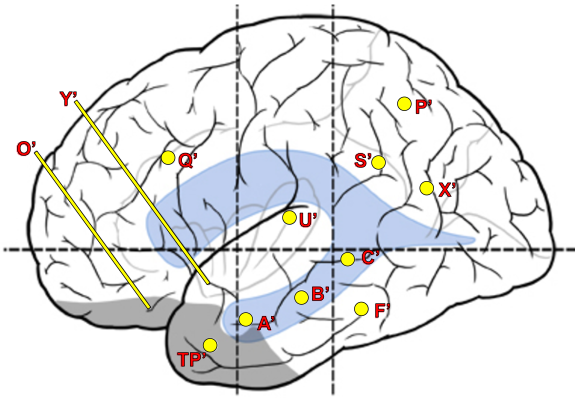
8	R	3	Right temporal	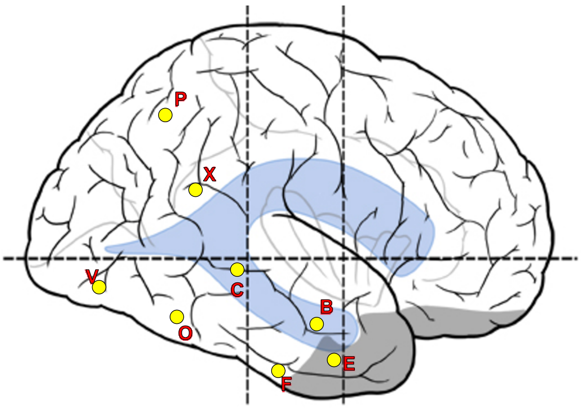

Subject	*Handedness*	*Duration*	*EZ*	*Electrode coverage*

Table S1: Table of each subject's number, handedness, duration of epilepsy (in years), Epileptogenic Zone (EZ), and electrode coverage. Electrode placement are approximated by the location of the lines or dots on the illustrated brain. Most electrodes were implanted laterally to medially, which are represented by a dot. Electrodes that were implanted sagittally are represented as a solid line. Each electrode was assigned with an alphabetical letter. Letters without an apostrophe mark electrodes that were implanted in the right hemisphere and letters with an apostrophe mark electrodes that were implanted in the left hemisphere. Yellow dots mark electrodes that were implanted in the hemisphere shown and green dots mark electrodes that were implanted in the opposite hemisphere than what is shown.

1.2 Figures

REFERENCES

Mori, S., Wakana, S., Zijl, P. C. V., and Nagae-Poetscher, L. M. (2005). *MRI atlas of human white matter* (Elsevier)

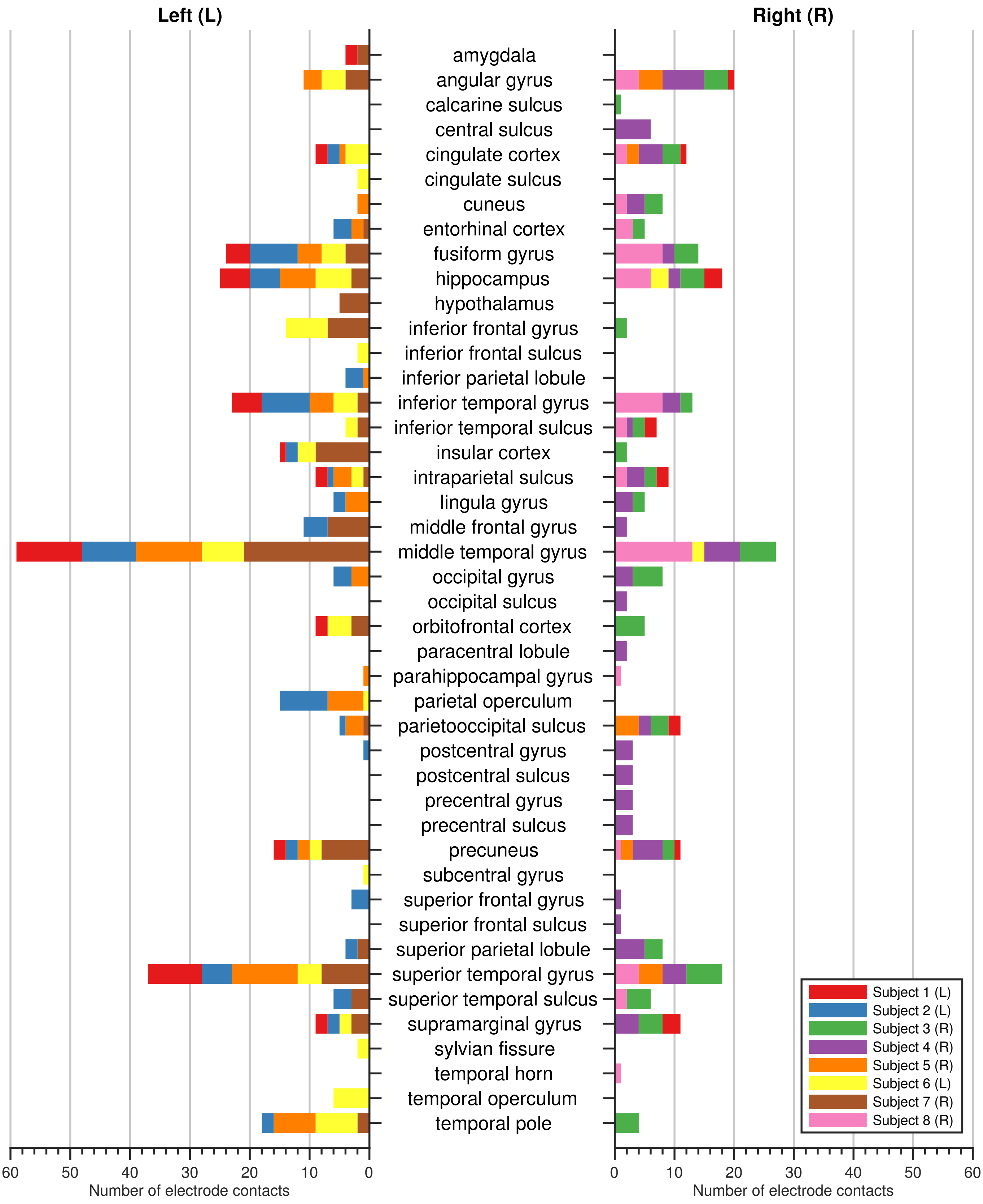

Figure S1: Complete list of electrode contact coverage across all subjects split into hemisphere of implantation. Contacts implanted in the left hemisphere are plotted on the left plot and right contacts are plotted on the right. Each subject is denoted as a different color bar. Length of each colored bar represents the number of contacts subject has in specified brain region.

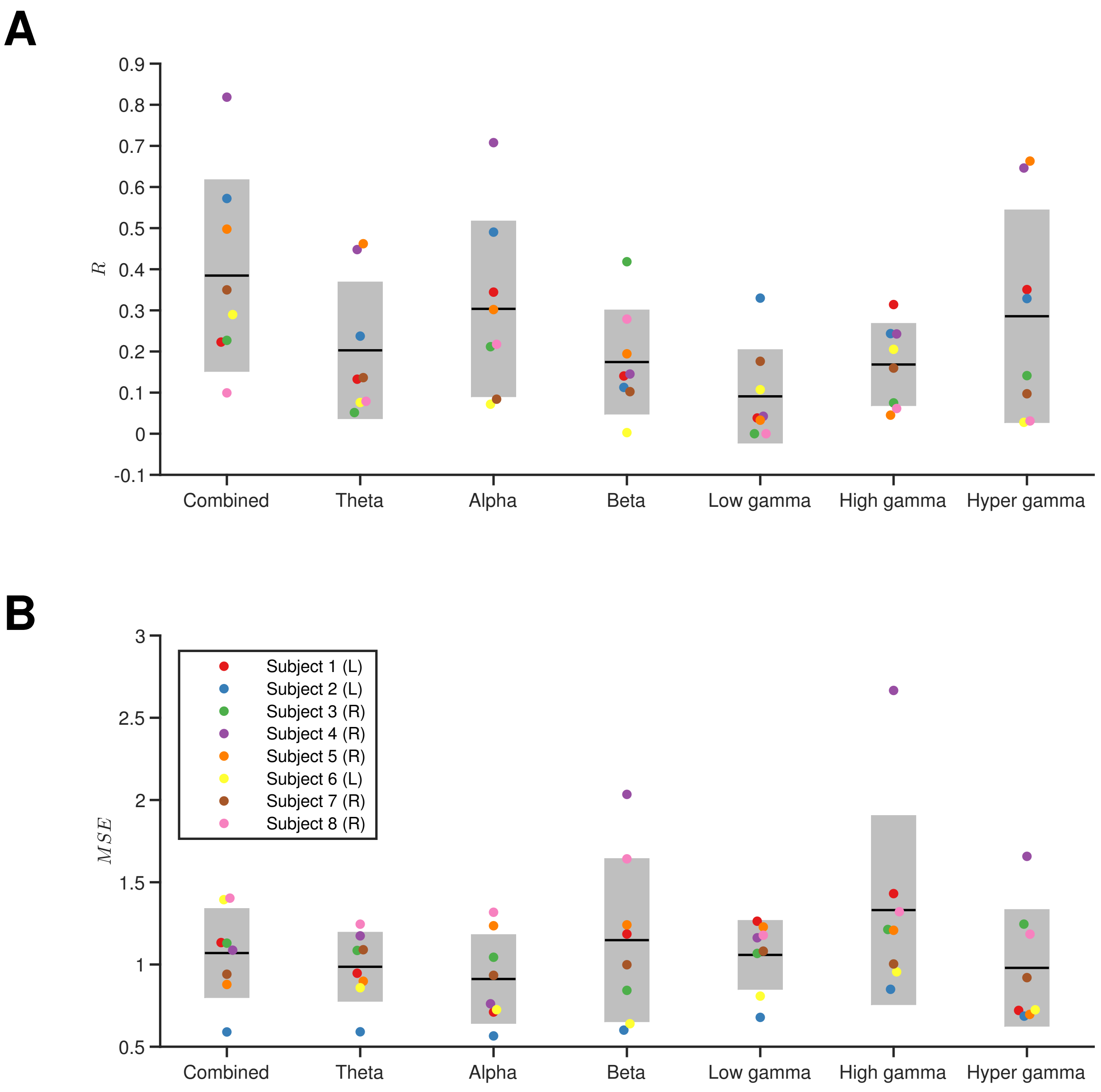

Figure S2: **(A)** The average correlation coefficient R between the actual speed and predicted speed using each frequency band model across 100 iterations on the test set for eight subjects. Each subject is denoted as a different color point. The mean of the metric is represented by the black line and one standard deviation is represented by the gray rectangle. **(B)** The average MSE between the actual speed and predicted speed using each frequency band model across 100 iterations on the test set for eight subjects. Each subject is denoted as a different color point. The mean of the metric is represented by the black line and one standard deviation is represented by the gray rectangle.

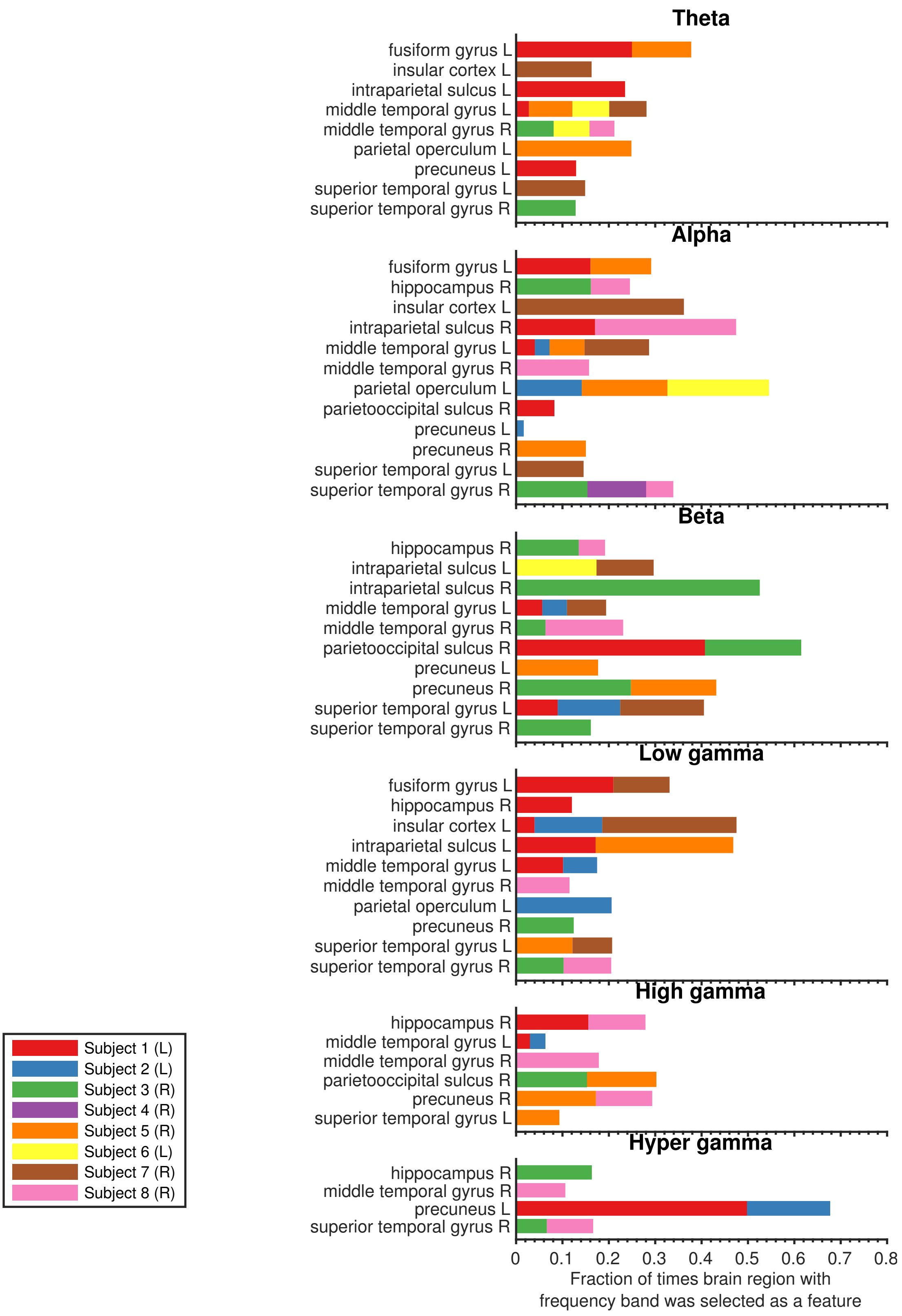

Figure S3: Break down of frequency bands selected as features in the combined model for eight subjects across 100 iterations. Length of each colored bar represents the number of features in the same frequency band was found relative to the total number of times feature label was selected across all subjects Only the features that were selected in the final model in at least associated with Figure 5 are shown. Features were then divided by frequencies to demonstrate which bands were selected in the combined model.

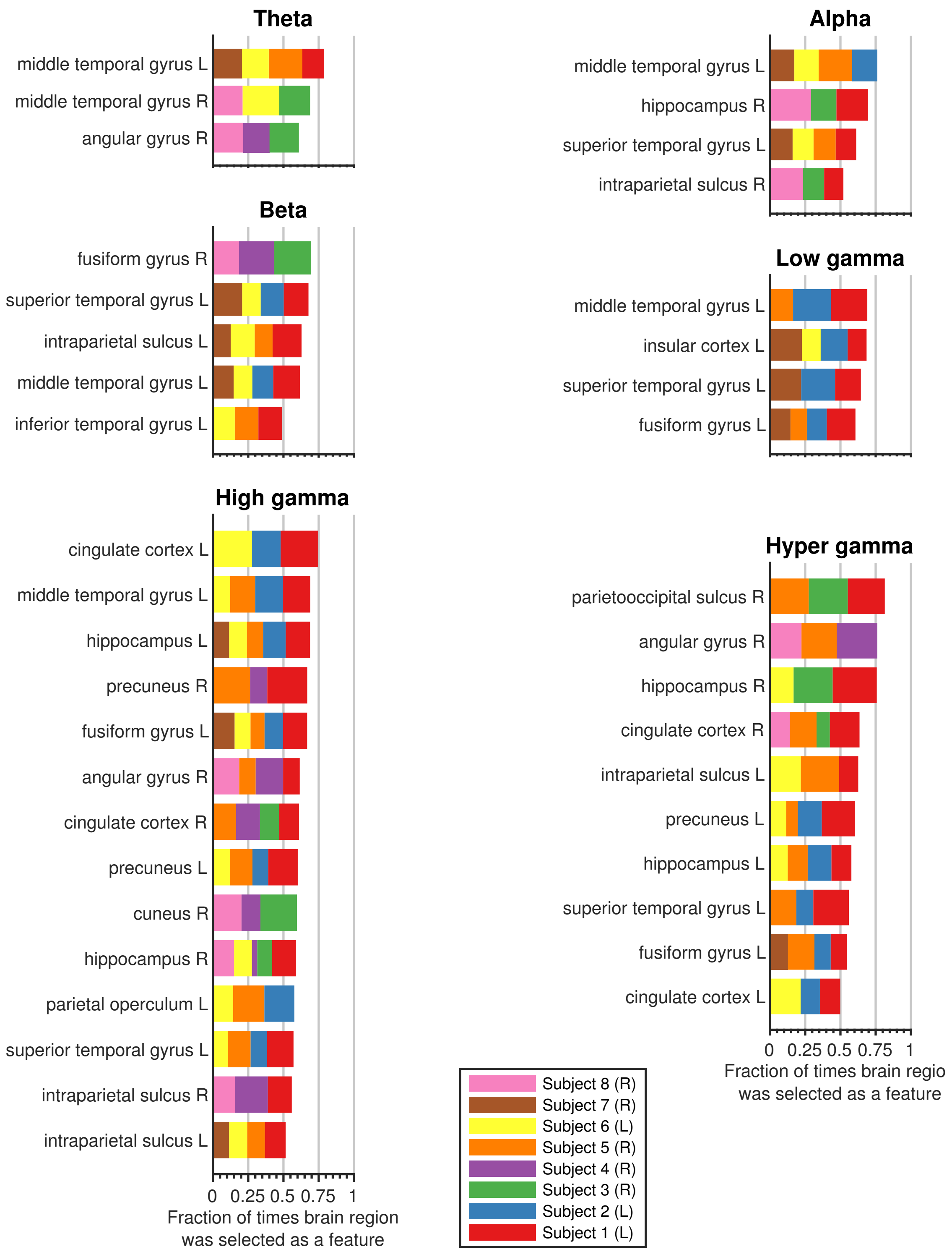

Figure S4: Stability of features in the six frequency band models for eight subjects, sorted by the fraction of times feature was picked during feature selection relative to the 100 iterations during 10-fold cross-validation. Each subject is denoted as a different color bar. Length of each colored bar represents the fraction of feature selected relative to the total number of times sampled within each subject. Only features that were selected as a final feature are shown.

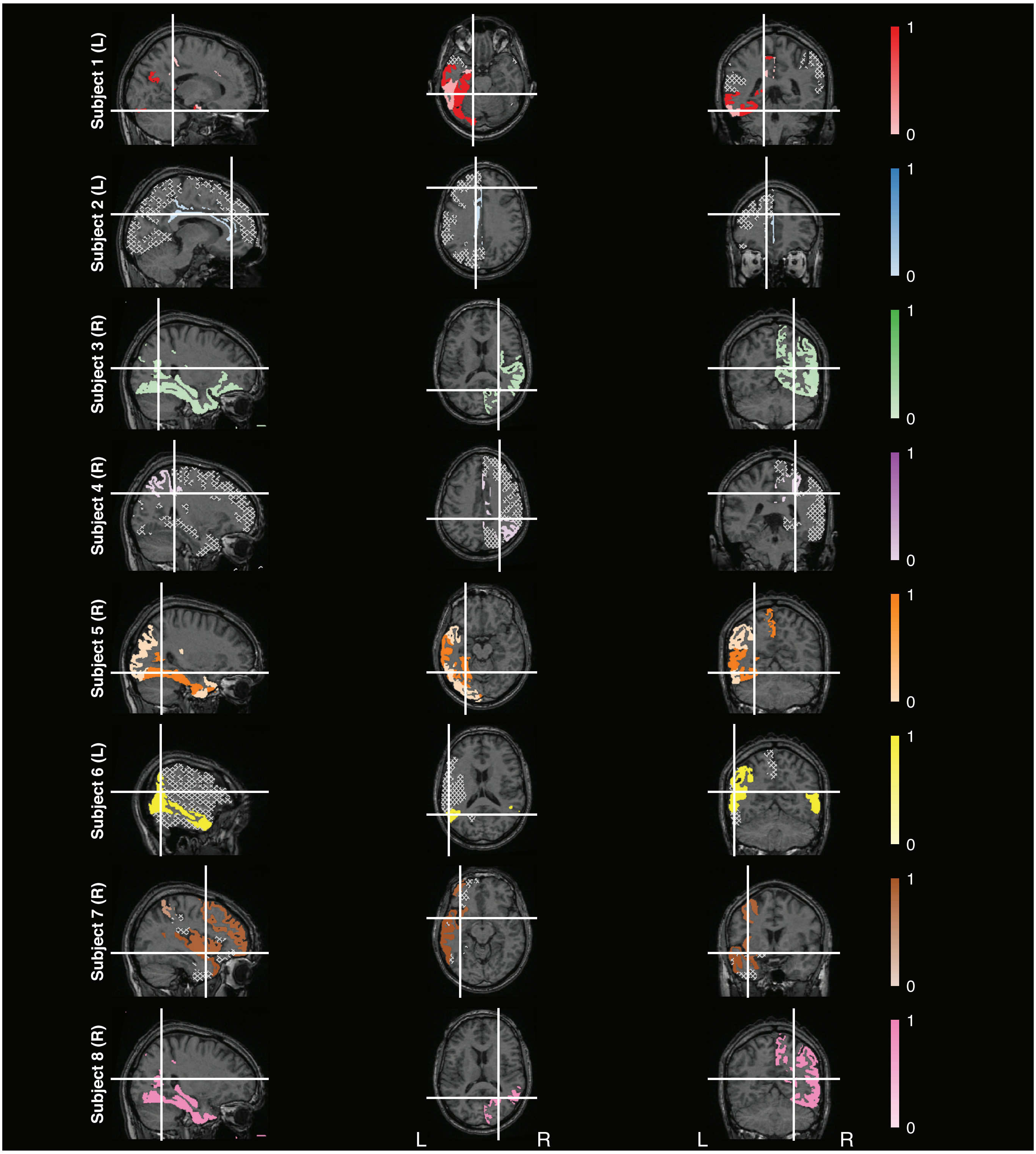

Figure S5: Map of features selected for the final theta model found using the test data for eight subjects, with subject numbers labeled vertically and handedness in parentheses. The white lines in each slice represents at which slice in the other viewpoints were taken. Electrode contacts selected as features were matched to labels from the Mori atlas (Mori et al., 2005). Brain regions that matched the feature labels were then highlighted on the corresponding MRI template used by the atlas. Regions were tinted based on how frequently it was selected as a final feature over 100 iterations, where regions close to zero (i.e., rarely selected as a final feature) are tinted whiter and regions close to one (i.e., often selected as a final feature) retain the original color. Not all features could be matched to an atlas label. Regions not selected as a final feature are filled in using a hatched grey area. These maps do not represent fMRI signals nor do they represent the exact location of the electrodes.

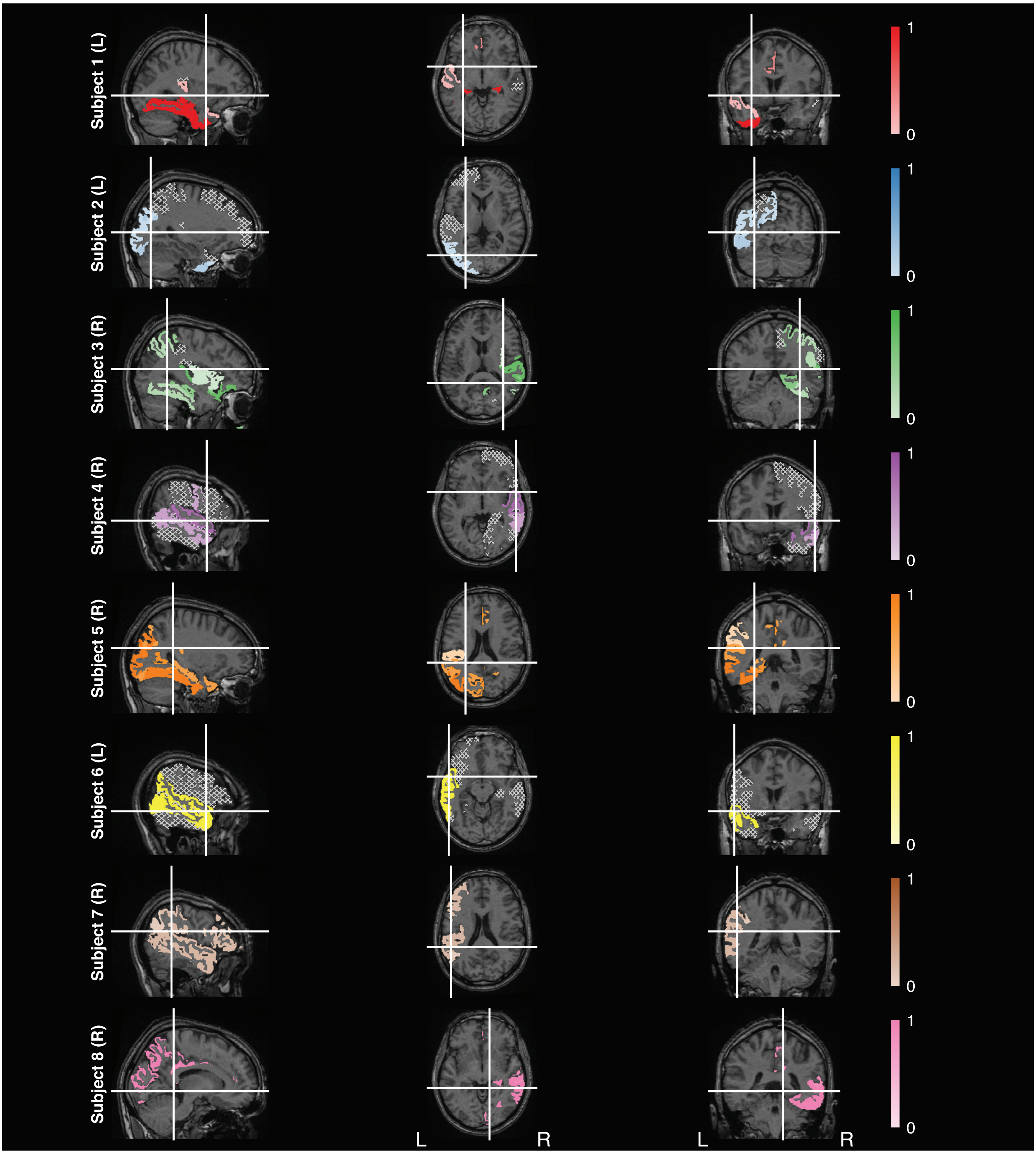

Figure S6: Map of features selected for the final alpha model found using the test data for eight subjects, with subject numbers labeled vertically and handedness in parentheses. The white lines in each slice represents at which slice in the other viewpoints were taken. Electrode contacts selected as features were matched to labels from the Mori atlas (Mori et al., 2005). Brain regions that matched the feature labels were then highlighted on the corresponding MRI template used by the atlas. Regions were tinted based on how frequently it was selected as a final feature over 100 iterations, where regions close to zero (i.e., rarely selected as a final feature) are tinted whiter and regions close to one (i.e., often selected as a final feature) retain the original color. Not all features could be matched to an atlas label. Regions not selected as a final feature are filled in using a hatched grey area. These maps do not represent fMRI signals nor do they represent the exact location of the electrodes.

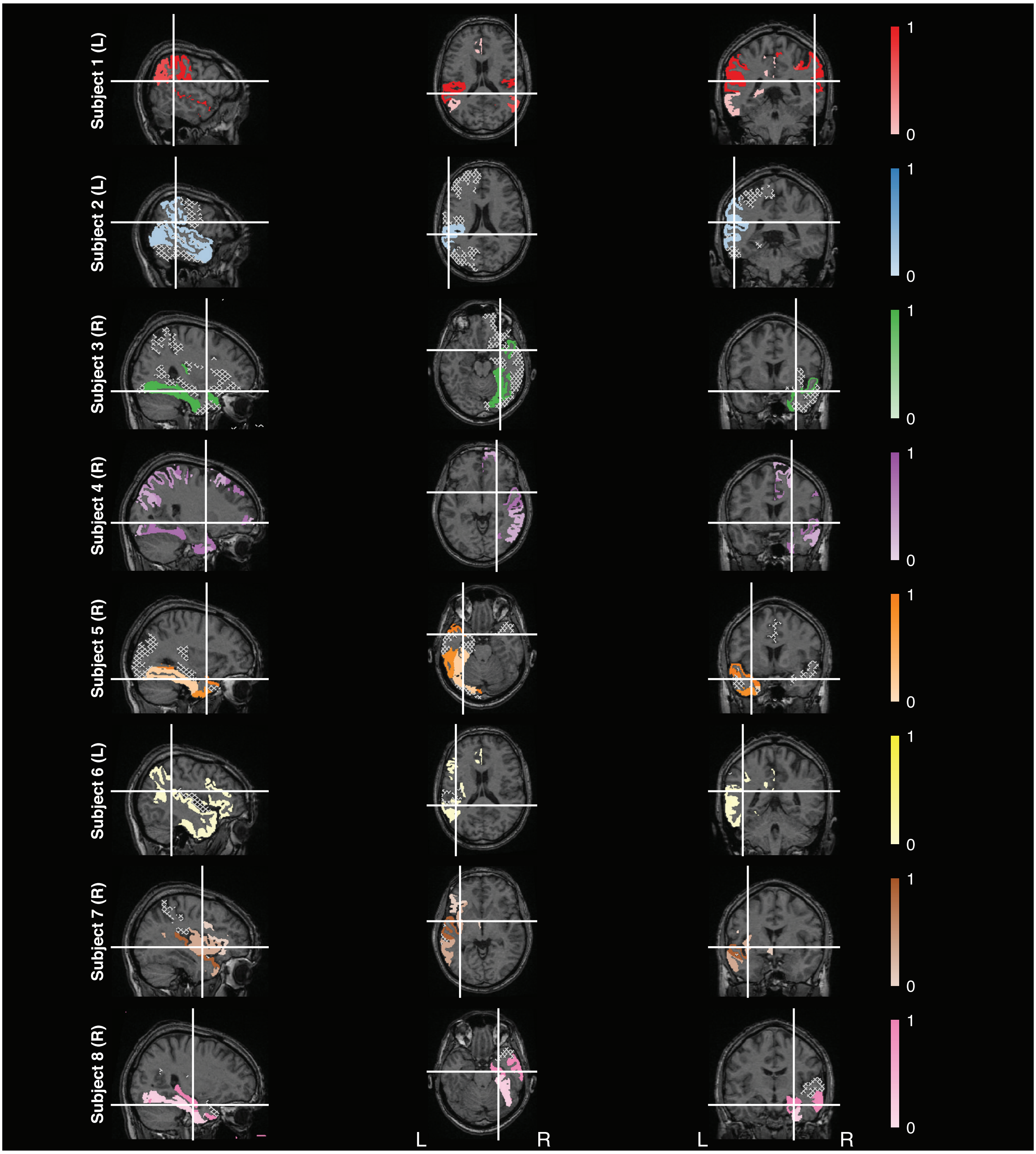

Figure S7: Map of features selected for the final beta model found using the test data for eight subjects, with subject numbers labeled vertically and handedness in parentheses. The white lines in each slice represents at which slice in the other viewpoints were taken. Electrode contacts selected as features were matched to labels from the Mori atlas (Mori et al., 2005). Brain regions that matched the feature labels were then highlighted on the corresponding MRI template used by the atlas. Regions were tinted based on how frequently it was selected as a final feature over 100 iterations, where regions close to zero (i.e., rarely selected as a final feature) are tinted whiter and regions close to one (i.e., often selected as a final feature) retain the original color. Not all features could be matched to an atlas label. Regions not selected as a final feature are filled in using a hatched grey area. These maps do not represent fMRI signals nor do they represent the exact location of the electrodes.

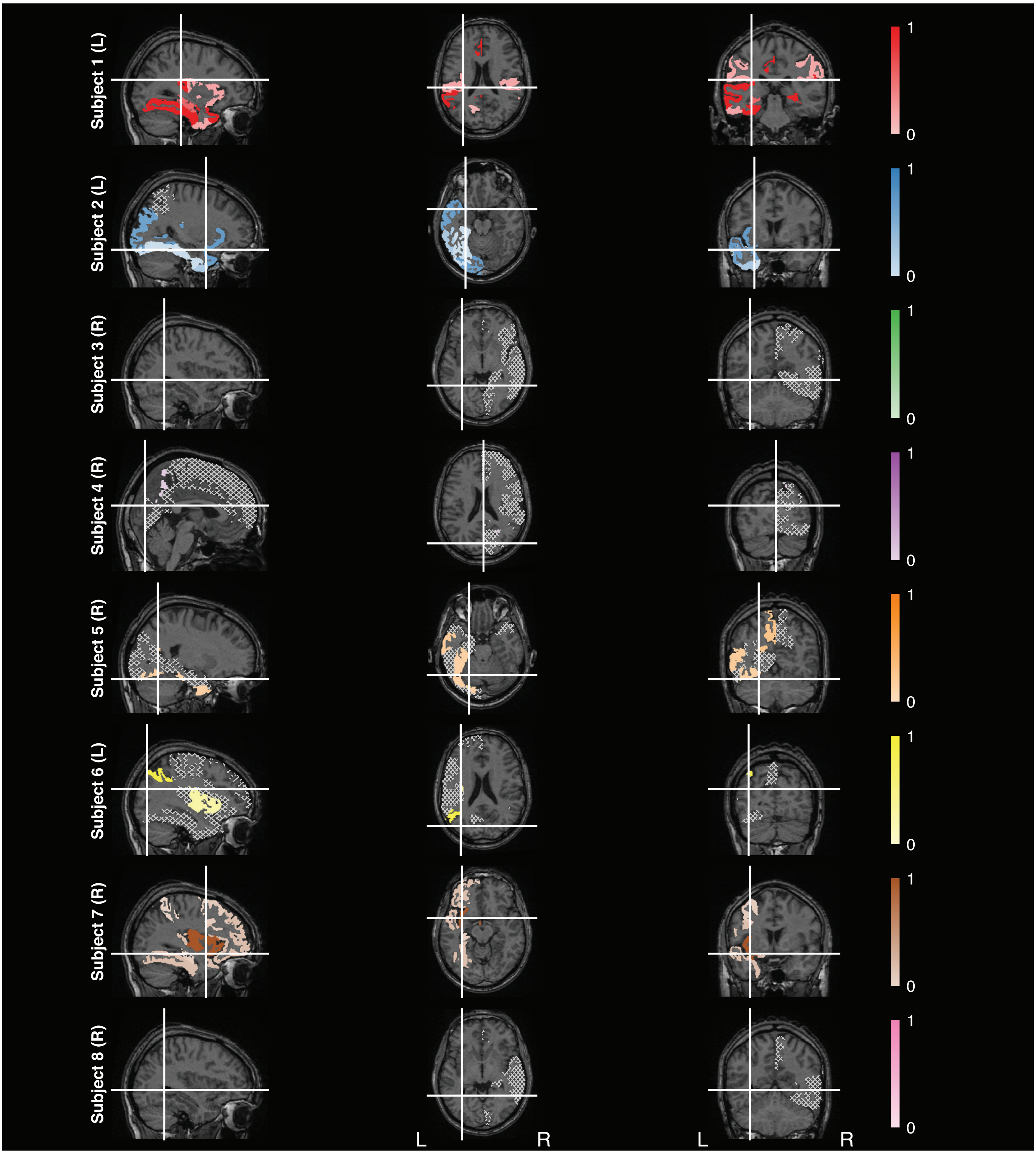

Figure S8: Map of features selected for the final low gamma model found using the test data for eight subjects, with subject numbers labeled vertically and handedness in parentheses. The white lines in each slice represents at which slice in the other viewpoints were taken. Electrode contacts selected as features were matched to labels from the Mori atlas (Mori et al., 2005). Brain regions that matched the feature labels were then highlighted on the corresponding MRI template used by the atlas. Regions were tinted based on how frequently it was selected as a final feature over 100 iterations, where regions close to zero (i.e., rarely selected as a final feature) are tinted whiter and regions close to one (i.e., often selected as a final feature) retain the original color. Not all features could be matched to an atlas label. Regions not selected as a final feature are filled in using a hatched grey area. These maps do not represent fMRI signals nor do they represent the exact location of the electrodes.

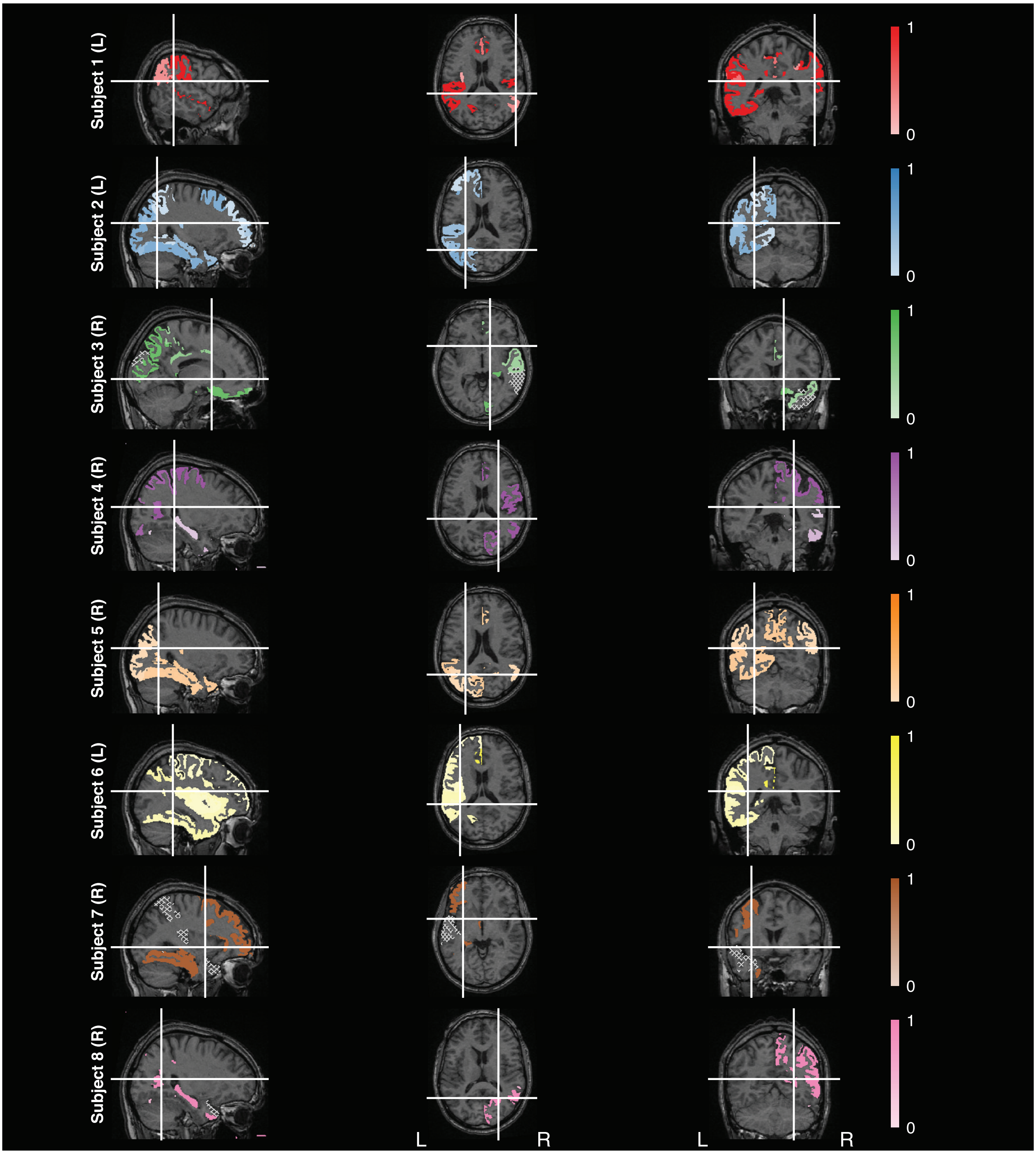

Figure S9: Map of features selected for the final high gamma model found using the test data for eight subjects, with subject numbers labeled vertically and handedness in parentheses. The white lines in each slice represents at which slice in the other viewpoints were taken. Electrode contacts selected as features were matched to labels from the Mori atlas (Mori et al., 2005). Brain regions that matched the feature labels were then highlighted on the corresponding MRI template used by the atlas. Regions were tinted based on how frequently it was selected as a final feature over 100 iterations, where regions close to zero (i.e., rarely selected as a final feature) are tinted whiter and regions close to one (i.e., often selected as a final feature) retain the original color. Not all features could be matched to an atlas label. Regions not selected as a final feature are filled in using a hatched grey area. These maps do not represent fMRI signals nor do they represent the exact location of the electrodes.

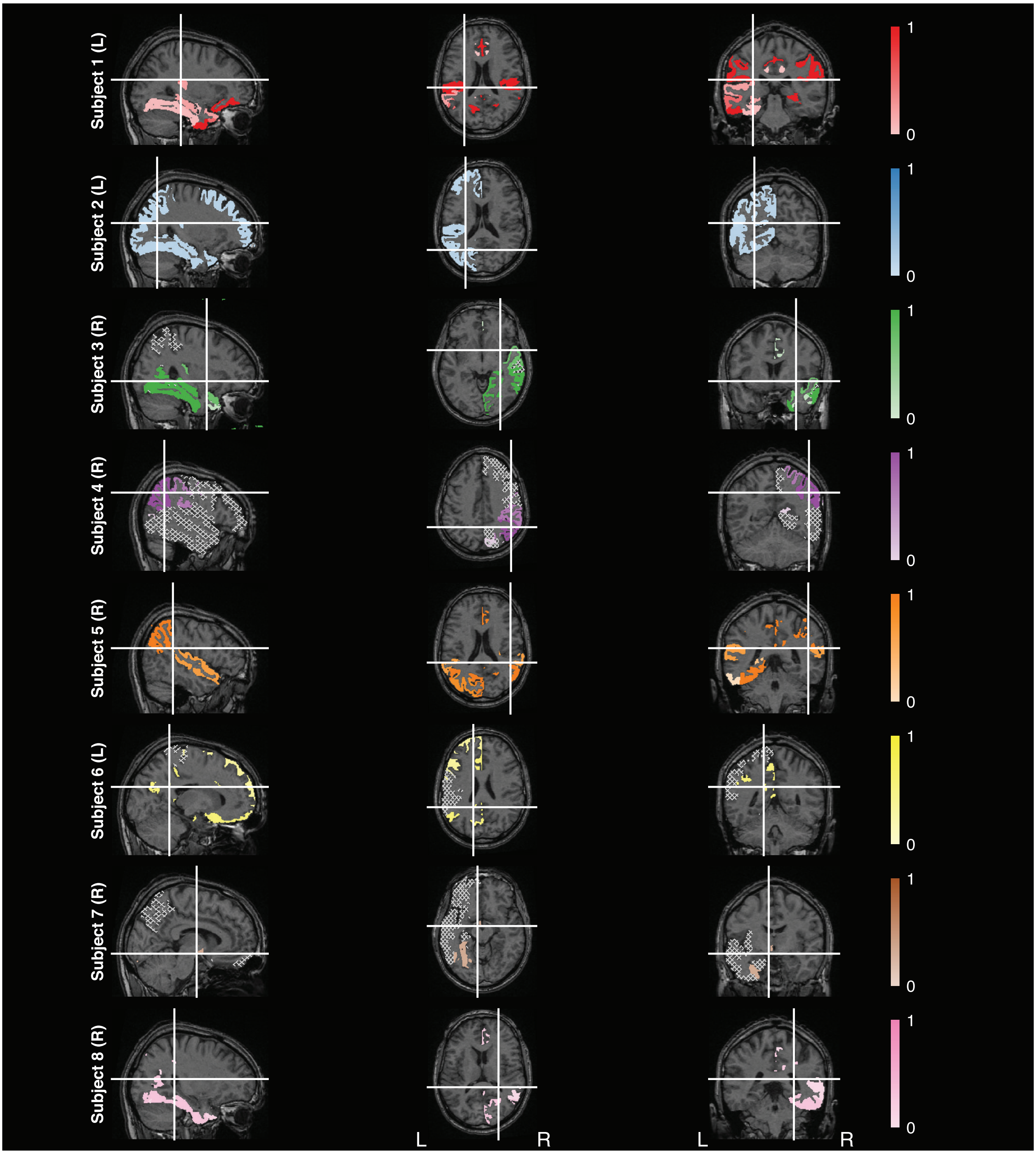

Figure S10: Map of features selected for the final hyper gamma model found using the test data for eight subjects, with subject numbers labeled vertically and handedness in parentheses. The white lines in each slice represents at which slice in the other viewpoints were taken. Electrode contacts selected as features were matched to labels from the Mori atlas (Mori et al., 2005). Brain regions that matched the feature labels were then highlighted on the corresponding MRI template used by the atlas. Regions were tinted based on how frequently it was selected as a final feature over 100 iterations, where regions close to zero (i.e., rarely selected as a final feature) are tinted whiter and regions close to one (i.e., often selected as a final feature) retain the original color. Not all features could be matched to an atlas label. Regions not selected as a final feature are filled in using a hatched grey area. These maps do not represent fMRI signals nor do they represent the exact location of the electrodes.
